# Actions and Mechanisms of Polyunsaturated Fatty Acids on Voltage-Gated Ion Channels

**DOI:** 10.3389/fphys.2017.00043

**Published:** 2017-02-06

**Authors:** Fredrik Elinder, Sara I. Liin

**Affiliations:** Department of Clinical and Experimental Medicine, Linköping UniversityLinköping, Sweden

**Keywords:** voltage-gated ion channels, polyunsaturated fatty acids, voltage sensor domain, S4, Excitability disorders

## Abstract

Polyunsaturated fatty acids (PUFAs) act on most ion channels, thereby having significant physiological and pharmacological effects. In this review we summarize data from numerous PUFAs on voltage-gated ion channels containing one or several voltage-sensor domains, such as voltage-gated sodium (Na_V_), potassium (K_V_), calcium (Ca_V_), and proton (H_V_) channels, as well as calcium-activated potassium (K_Ca_), and transient receptor potential (TRP) channels. Some effects of fatty acids appear to be channel specific, whereas others seem to be more general. Common features for the fatty acids to act on the ion channels are at least two double bonds in *cis* geometry and a charged carboxyl group. In total we identify and label five different sites for the PUFAs. *PUFA site 1*: The intracellular cavity. Binding of PUFA reduces the current, sometimes as a time-dependent block, inducing an apparent inactivation. *PUFA site 2*: The extracellular entrance to the pore. Binding leads to a block of the channel. *PUFA site 3*: The intracellular gate. Binding to this site can bend the gate open and increase the current. *PUFA site 4*: The interface between the extracellular leaflet of the lipid bilayer and the voltage-sensor domain. Binding to this site leads to an opening of the channel via an electrostatic attraction between the negatively charged PUFA and the positively charged voltage sensor. *PUFA site 5*: The interface between the extracellular leaflet of the lipid bilayer and the pore domain. Binding to this site affects slow inactivation. This mapping of functional PUFA sites can form the basis for physiological and pharmacological modifications of voltage-gated ion channels.

## Introduction

Fish, fish oils, and polyunsaturated fatty acids (PUFAs; which are major components of fish oils) have beneficial effects on cardiac-, brain-, and muscle-related disorders. This has been shown in a number of studies at different levels:

1. Anthropological studies suggest that the Eskimo and Mediterranean diets, rich in mono- and PUFAs, lower the risk of heart disease and early death (Keys, 1970; Bang et al., 1971) (but see Fodor et al., 2014).
2. Large clinical trials show beneficial effects of dietary fish oil or PUFAs with decreased risk of sudden cardiac death (Burr et al., 1989; de Lorgeril et al., 1994; GISSI-Prevenzione Investigators, 1999; Albert et al., 2002; Marchioli et al., 2002).
3. *In vivo* animal models show that both intraperitoneal and intravenous administration of fish oil or isolated PUFAs prevent induced fatal ventricular arrhythmias (McLennan et al., 1988; McLennan, 1993; Billman et al., 1994, 1997, 1999).
4. *In vitro* models show that PUFAs applied directly to cardiomyocytes terminate arrhythmia and arrhythmia resumes upon removal of PUFAs (Kang and Leaf, 1994).

The last point suggests that PUFAs merely need to partition into the phospholipid cell membrane to exert their antiarrhythmic effect, probably via ion channels, which are responsible for electrical excitability of cells. Despite intense research, the molecular details of the action of PUFAs on ion channels and on excitability are largely unknown. In this review we will summarize what is known about the interaction between PUFAs and one superfamily of ion channels, the voltage-gated ion channels.

Voltage-gated ion channel are pore-forming molecules in the lipid bilayer of most cells, which open in response to alterations in the cell's transmembrane electrical potential (Hille, 2001). Opening of these channels allows the passage of specific types of ion across the cell membrane, thereby initiating and altering essential processes such as, signaling via nervous impulses, or movement via muscle contractions. Ion channels can be regulated by endogenous or exogenous compounds like hormones, pharmaceutical drugs, or toxins. Some compounds, such as PUFAs, can be both endogenous and exogenous.

PUFA effects on ion channels have been reviewed in several excellent papers (Ordway et al., 1991; Meves, 1994; Leaf and Xiao, 2001; Boland and Drzewiecki, 2008) but few, if any, have tried to outline the molecular sites of action and the molecular mechanism of the effects. Even fewer have tried to search for common mechanisms across the channel families. These two aspects are the focus of the present review. We will start with brief overviews of voltage-gated ion channels and of PUFAs. Then, we will summarize the current literature concerning PUFA effects on voltage-gated ion channels. This will be followed by an attempt to explain the data in molecular terms. Finally, we will briefly discuss relevant physiological and therapeutic implications.

## The superfamily of voltage-gated ion channels

The general structure of voltage-gated ion channels has been described in many extensive reviews (e.g., Tombola et al., 2006; Catterall et al., 2007; Bezanilla, 2008; Börjesson and Elinder, 2008). Therefore, we will only briefly describe core features that are pertinent to the subsequent discussion.

**Figure 1:**
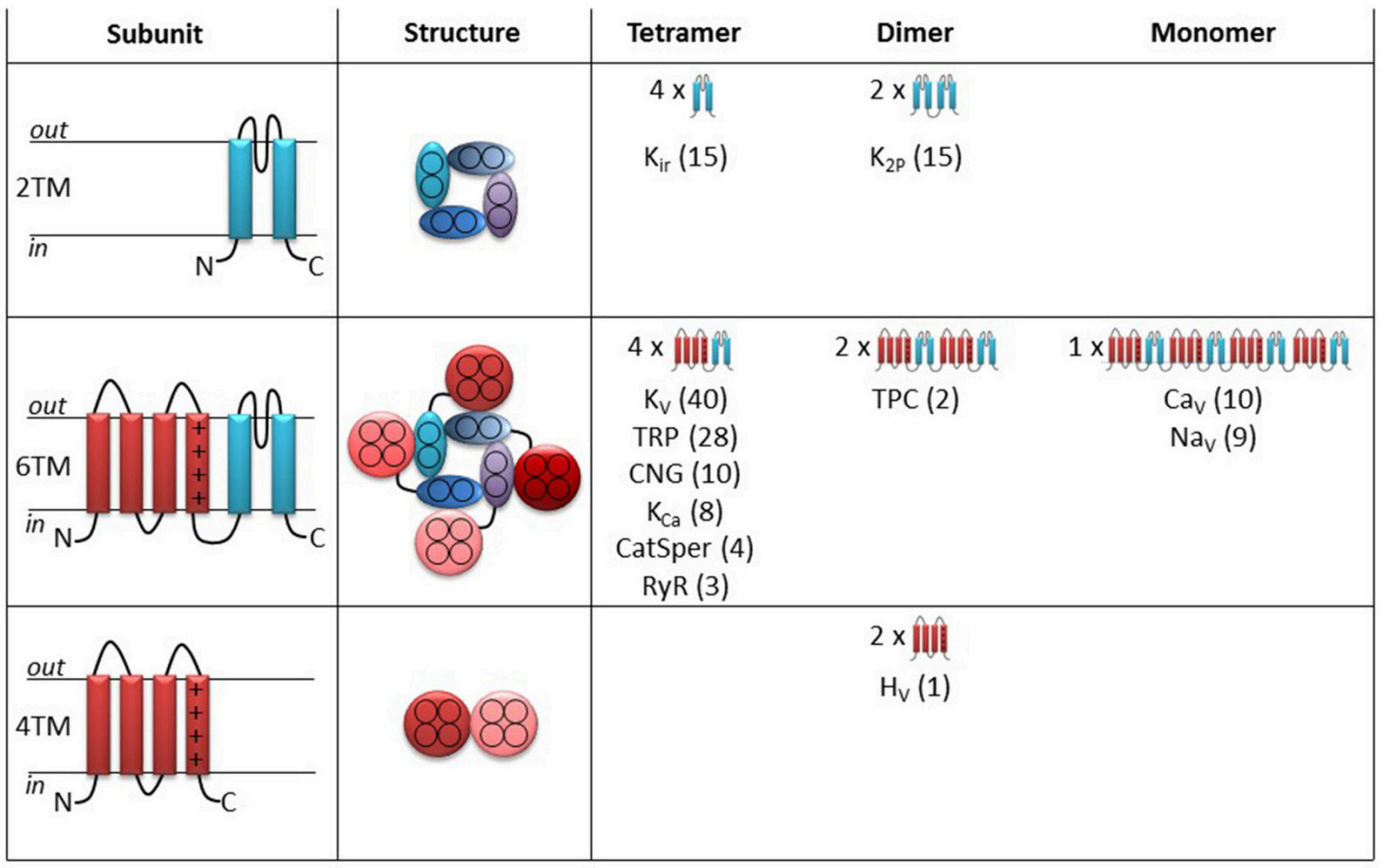
**Topology and cartoons on the different ion channels in the superfamily of voltage-gated ion channels**. Left column illustrates side view of the topology of a single subunit. Pore forming segments in blue and voltage-sensor domain segments in red. Middle column illustrates top view of the functional ion channel. Right column provides an overview of the different subfamilies and their topology. The numbers in parentheses denote the number of ion channels within each subfamily.

The human genome contains 144 genes coding for members of the superfamily of voltage-gated ion channels (http://guidetopharmacology.org/GRAC/ReceptorFamiliesForward?type=IC). Figure 1 shows an overview of how these 144 channels are classified into families.

Thirty of the channels (upper row in Figure 1) only contain pore-forming subunits (blue in Figure 1). Each pore-forming subunit has two transmembrane (2TM) segments with a pore-lining segment in-between (left column in Figure 1). Four pore-forming subunits fused together make up a functional channel with a central ion-conducting pore (middle column). This tetrameric structure is referred to as the *pore domain*. The potassium-selective inward rectifiers (K_ir_) are examples of such channels (Figure 1, right column). Also the two-pore potassium (K_2P_) channels have a similar 3D architecture but are instead formed as dimer-of-dimers (each K_2P_ gene is coding for two linked pore-forming subunits). Channels that contain only the pore domain are not intrinsically voltage sensitive but belong to the superfamily of voltage-gated ion channels because of molecular kinship. These channels are, instead, regulated by mechanical forces or ligands (Kim, 2003; Honoré, 2007).

113 channels in the superfamily of voltage-gated ion channels are composed of pore-forming segments, as described above, linked to voltage sensing segments (red in Figure 1) in a six transmembrane (6TM) architecture (Figure 1, middle row). These types of channels have a central pore domain surrounded by four *voltage-sensor domains* (VSDs) (Figure 1, middle column, middle row). In most cases, the VSD confers voltage dependence to these channels. Molecular details about the voltage-sensing mechanism will be described below when we discuss the molecular mechanism for PUFA action on voltage-gated ion channels. Six families are arranged as tetramers of 6TM subunits (Figure 1, right column, middle row): Voltage-gated K (K_V_) channels, transient receptor potential (TRP) channels, cyclic nucleotide activated (CNG) channels (including the hyperpolarization and cyclic nucleotide-activated (HCN) channels), calcium-activated K (K_Ca_) channels, ryanodine receptors (RyR), and cation channels of sperm (CatSper). In contrast, two-pore (TPC) channels are formed as dimers of two linked 6TM subunits, while voltage-gated calcium (Ca_V_) and sodium (Na_V_) channels are formed as monomers of four linked 6TM subunits.

Finally, one channel, the voltage-gated proton (H_V_1) channel is a dimer of 4TM-VSD motifs (Figure 1, lower row). This channel lacks the pore domain but allows protons to pass through the center of each VSD (Koch et al., 2008; Tombola et al., 2008).

The present review focuses on PUFA effects on intrinsically voltage-gated ion channels. We will therefore mainly summarize and discuss data from the VSD-containing channels (6TM and 4TM channels in Figure 1, the middle and lower rows). Effects on the channels in the upper row will not be covered. However, some of the 2TM channels are highly sensitive to PUFAs, such that some of them have names reflecting regulation by PUFAs. For example, the K_2P_4.1 channel is also referred to as the TWIK-related *arachidonic-acid* activated K (TRAAK) channel. Some of the described PUFA effects on these channels will be briefly mentioned later in this review, when we discuss the molecular mechanism of PUFA effects on intrinsically voltage sensitive ion channels. It should also be noted that some early studies were performed before the molecular identity was known. In these cases we have assigned channels to different families based of their functional characteristics.

## Classification and sources of fatty acids

**Figure 2:**
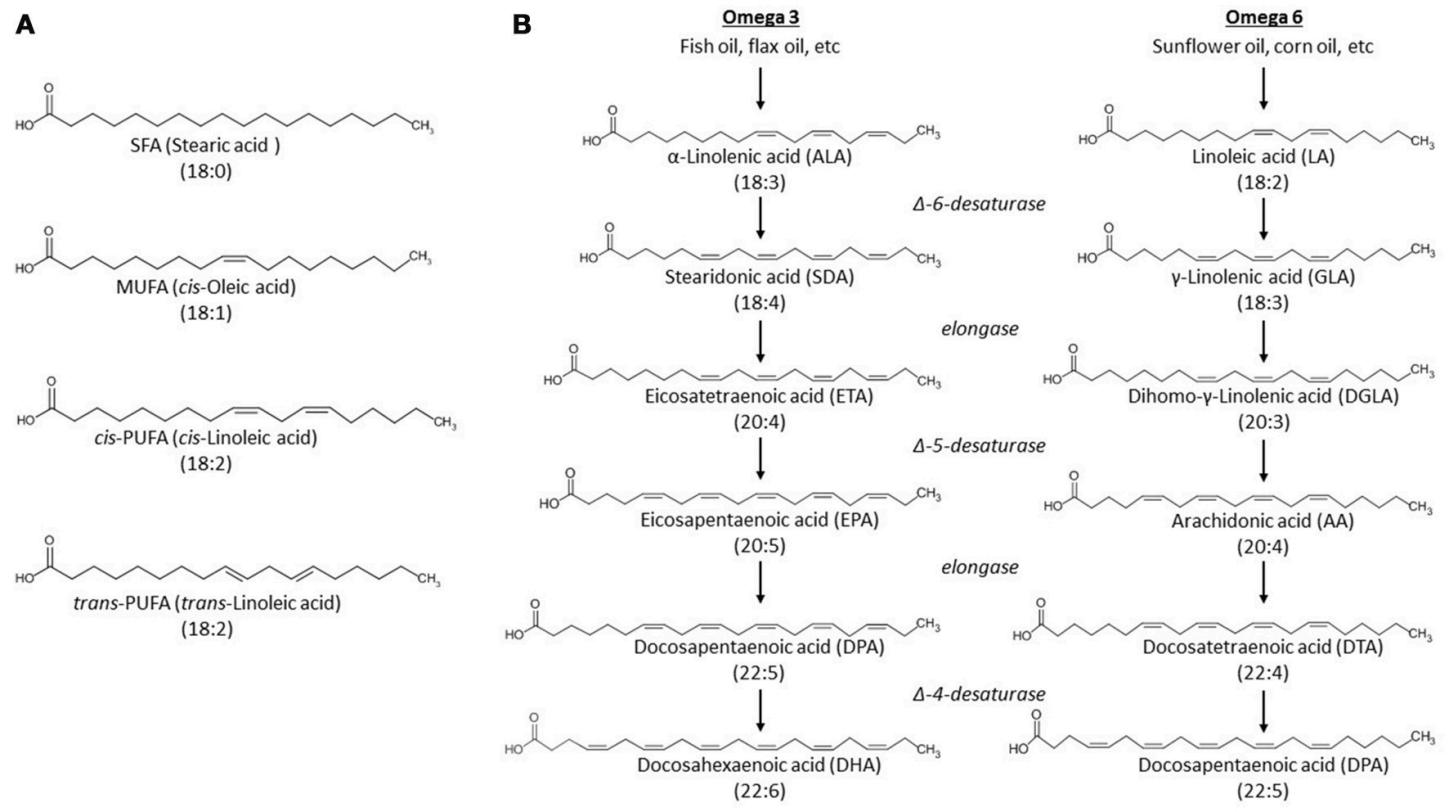
**Structures of unesterified fatty acids. (A)** Unesterified fatty acids are classified according to the presence, number and geometry of double bonds in the acyl tail. Abbreviations: MUFA, monounsaturated fatty acid; SFA, saturated fatty acid; PUFA, polyunsaturated fatty acid; The PUFA is shown in both *cis* and *trans* geometry. **(B)** Metabolic pathways of n-3 and n-6 fatty acid synthesis. α-linolenic acid and linoleic acid are the precursors of n-3 and 6 PUFAs, respectively. Different desaturases and elongases convert these precursors to different long-chain PUFAs.

Fatty acids are important messengers in cell signaling and critical components of the phospholipids that constitute the plasma membrane. The general structure of most naturally occurring fatty acids is a carboxylic acid with an unbranched aliphatic hydrocarbon tail. These fatty acids can be classified according to the number of carbon-carbon double bonds in the tail (Figure 2A):

– Saturated fatty acids (SFAs) such as stearic acid lack double bonds.
– Monounsaturated fatty acids (MUFAs) such as oleic acid have one double bond.
– Polyunsaturated fatty acids (PUFAs) such as linoleic acid, arachidonic acid (AA), and docosahexaenoic acid (DHA) have two or more double bonds.

A common way to name fatty acids is by the number of carbons and double bonds. For example, DHA is also called 22:6 (22 carbons and six double bonds). Moreover, double bonds can display *cis* geometry (the adjacent carbons are on the same side of the carbon chain) or *trans* geometry (the adjacent carbons are on opposite sides of the carbon chain). *Cis* geometry is most common among naturally occurring unsaturated fatty acids, while *trans* is usually caused by industrial processing of fatty acids (Micha and Mozaffarian, 2009) (Figure 2A).

Certain fatty acids, in particular SFAs and MUFAs, can be synthesized *de novo* in the human body (Mullen and Yet, 2015). Others, especially PUFAs, must instead be acquired through the diet (Jakobsson et al., 2006; Kihara, 2012). Dietary intake of α-linolenic acid and linoleic acid (obtained from fish oil or sunflower oil, respectively) is a vital source for PUFAs (Figure 2B). The first double bond in α-linolenic acid is located at the third carbon, counting from the methyl end of the tail, and is therefore an n-3 (or ω-3) fatty acid. Linoleic acid, on the other hand, has its first double bond located at the sixth carbon, and is therefore an n-6 (or ω-6) fatty acid. These dietary PUFAs function as precursors in the synthesis of longer PUFAs like the n-3 docosahexaenoic acid (DHA) or the n-6 arachidonic acid (AA) (Figure 2B). Non-esterified fatty acids can circulate in the plasma bound to transport proteins such as albumin. These non-esterified free fatty acids are directly available to dissociate from albumin and interact with membrane-bound ion channels (as will be discussed later) or be metabolized by various enzymatic systems (described below).

**Figure 3:**
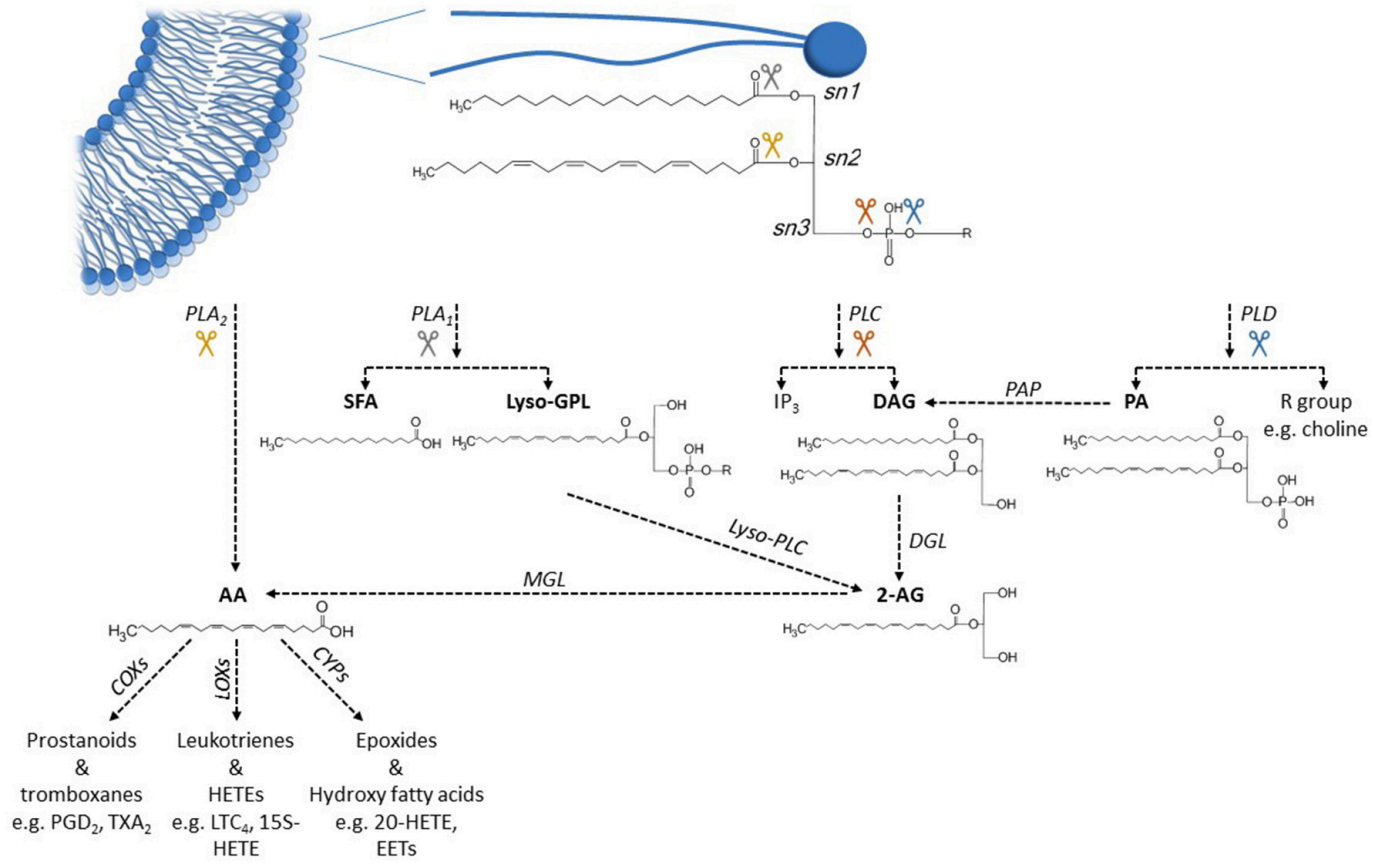
**Metabolic pathways of arachidonic acid hydrolysis and oxidation**. Phospholipids in the cell membranes commonly have a SFA esterified to *sn*1 position and a PUFA, such as arachidonic acid (AA) esterified to *sn*2 position. Activation of different phospholipases releases AA from phospholipids, either in one enzymatic step (PLA_2_) or through several enzymatic steps (PLA_1_, PLC, PLD). Unesterified AA can be further metabolized to various eicosanoid metabolites by different COX, LOX, and CYP enzymes. Abbreviations: 2-AG, 2-arachidonoylglycerol; AA, arachidonic acid; COX, cyclooxygenase; CYP, cytochrome P450 enzyme; DAG, 1,2-diacylglycerol; DGL, DAG lipase; EET, epoxyeicosatrienoic acid; HETE, hydroxyeicosatrienoic acid; IP_3_, inositol 1,4,5-trisphosphate; LOX, lipooxygenase; LTC_4_, leucotriene C_4_; Lyso-GPL, lyso-glycerolphospholipid; Lyso-PLC, lysophospholipase C; MGL, monoacylglycerol lipase; PA, phosphatidic acid; PAP, PA phosphatase; PGD_2_, prostaglandin D_2_; PLA_1_, phospholipase A_1_; PLA_2_, phospholipase A_2_; PLC, phospholipase C; PLD, phospholipase D; SFA, saturated fatty acid; TXA_2_, thromboxane A_2_.

The phospholipids that constitute the plasma membrane are another important source for fatty acids. Each phospholipid is composed of two fatty acids and a head-group bound to a glycerol backbone (Figure 3). SFAs are generally esterified to the first carbon of the glycerol backbone (*sn*1) while PUFAs, or (less commonly) MUFAs, are esterified to the second carbon (*sn*2). The polarity and charge of different phospholipids are determined by the properties of the head group bound to the third carbon of the glycerol backbone (*sn*3). Esterified fatty acids in the plasma membrane can be hydrolyzed to non-esterified free fatty acids, which are then available to interact with ion channels and other cellular proteins. The hydrolysis of esterified AA has been most extensively studied. It is primarily mediated by four different phospholipases that act at four distinct sites in the phospholipid (Figure 3) (Dennis et al., 1991; Siddiqui et al., 2008); Phospholipase A_2_ (PLA_2_) -mediated hydrolysis of the *sn*2 linkage directly releases AA. In contrast, Phospholipase A_1_ (PLA_1_), phospholipase C (PLC), or phospholipase D (PLD) -mediated hydrolysis yield precursors of AA (such as 1, 2 diacylglycerol and phosphatidic acid) that require additional enzymatic conversions before non-esterified AA is released. AA and DHA are the most common PUFAs to be found in *sn*2 position in mammalian phospholipids. Release of DHA (or other unsaturated fatty acids) from phospholipids follows the same overall pathway as AA release, although the chemical intermediates formed are different due to differences in the fatty acid acyl tail.

Once released from the plasma membrane, these non-esterified fatty acids may diffuse to and interact with membrane-bound ion channels, take part in intracellular signaling, or be further metabolized by various oxygenases. Metabolism of non-esterified fatty acids is mediated by three main types of oxygenases (Figure 3) (Siddiqui et al., 2008; Jenkins et al., 2009): Cyclooxygenases (COX), lipoxygenases (LOX), and cytochrome P450 epoxygenases (CYP). These enzymes produce a family of fatty-acid metabolites named eicosanoids, which includes prostaglandins, leukotrienes, thromboxanes, and epoxides (Siddiqui et al., 2008; Jenkins et al., 2009). Again, the structures of these metabolites depend on the structure of the specific fatty acid that is substrate for oxygenation.

In this review we will focus on the effect of non-esterified PUFAs on voltage-gated ion channels. Several fatty acid metabolites and intermediates formed during phospholipid hydrolysis are also known to modulate the activity of voltage-gated ion channels. However, we will not discuss these interactions here.

## Effects of PUFA on voltage-gated ion channels

To collect papers describing the effects of PUFA on the VSD-containing channels we searched PubMed for various combinations of voltage-gated ion channels and fatty acids, and extended the list when relevant articles were found during the work. In total we identified, read and analyzed data from 295 original papers containing voltage-clamp data from voltage-gated ion channels published between 1987 and June 2016 (Table 1). In addition, we read and analyzed about 400 papers concerning PUFA effects on non-VSD containing channels, review papers, or papers describing PUFA effects on excitability in general.

**Table 1:** **List of general effects and references to all articles analyzed in the present review**.

**Family**	**Amplitude**	**G(V)**	**ss-inact**.	**Inactivation**	**No articles**	**References**
K_V_1-4	↓	←	←	Faster	76	a
K_V_7	↑	←	–	–	9	b
K_V_10-12	↑↓	←	–	–	5	c
K_Ca_	↑	←	–	–	53	d
TRP	↑	–	–	–	24	e
CNG	↑↓	–	–	–	2	f
RyR	↑↓	–	–	–	3	g
Catsper/TCP	↑↓	–	–	–	4	h
Na_V_	↓	↔	←	–	41	i
Ca_V_	↓	←	←	Faster	69	j
H_V_	↑	←	–	–	8	k

*The arrows denote the general effect in each family. Double arrows denote mixed effects. A dash denote that the parameter has not been investigated, there is no effect, or that it is not applicable. a, (Takenaka et al., 1987, 1988; Premkumar et al., 1990; Rouzaire-Dubois et al., 1991; Damron et al., 1993; Villarroel, 1993; Chesnoy-Marchais and Fritsch, 1994; Honoré et al., 1994; Lee et al., 1994; Lynch and Voss, 1994; Gubitosi-Klug et al., 1995; Poling et al., 1995; Nagano et al., 1995a; Poling et al., 1996; Soliven and Wang, 1995; Wang and Lu, 1995; Nagano et al., 1997; Garratt et al., 1996; Smirnov and Aaronson, 1996; Villarroel and Schwarz, 1996; Gilbertson et al., 1997; Horimoto et al., 1997; Keros and McBain, 1997; Bogdanov et al., 1998; Bringmann et al., 1998; Devor and Frizzell, 1998; Dryer et al., 1998; Hatton and Peers, 1998; Visentin and Levi, 1998; Bittner and Müller, 1999; Colbert and Pan, 1999; Singleton et al., 1999; Yu et al., 1999; Casavant et al., 2000; Wilson et al., 2000; Holmqvist et al., 2001; Kehl, 2001; McKay and Jennings, 2001; Takahira et al., 2001; Erichsen et al., 2002; Müller and Bittner, 2002; Ramakers and Storm, 2002; Seebungkert and Lynch, 2002; Xiao et al., 2002; Danthi et al., 2003; Ferroni et al., 2003; Judé et al., 2003; Fioretti et al., 2004; Oliver et al., 2004; Sokolowski et al., 2004; Angelova and Müller, 2006, 2009; Feng et al., 2006; Kang et al., 2006; Jacobson et al., 2007; Szekely et al., 2007; Zhao et al., 2007; Börjesson et al., 2008, 2010; Guizy et al., 2008; Xu et al., 2008; Zhang M. et al., 2008; Boland et al., 2009; Koshida et al., 2009; Li et al., 2009; Wang et al., 2009; Decher et al., 2010; Börjesson and Elinder, 2011; Lai et al., 2011; Kong et al., 2012; Heler et al., 2013; Carta et al., 2014; Ottosson et al., 2014; Bai et al., 2015; Farag et al., 2016; Yazdi et al., 2016). b, (Béhé et al., 1992; Villarroel, 1993, 1994; Yu, 1995; Doolan et al., 2002; Milberg et al., 2011; Liin et al., 2015, 2016a,b; Moreno et al., 2015). c, (Schledermann et al., 2001; Liu and Wu, 2003; Wang et al., 2004; Guizy et al., 2005; Gavrilova-Ruch et al., 2007). d, (Bregestovski et al., 1989; Kirber et al., 1992; Ling et al., 1992; Ahn et al., 1994; Duerson et al., 1996; Zou et al., 1996; Twitchell et al., 1997; Devor and Frizzell, 1998; Stockand et al., 1998; Denson et al., 1999, 2000, 2005, 2006; Barlow et al., 2000; Wu et al., 2000; Fukao et al., 2001; Lu et al., 2001, 2005; Zhang et al., 2001; Zhang P. et al., 2008; Clarke et al., 2002, 2003; Lauterbach et al., 2002; Li et al., 2002, 2010; Ye et al., 2002; Hamilton et al., 2003; Gauthier et al., 2004, 2014; Zheng et al., 2005, 2008; Yang M. et al., 2005; Sun et al., 2007, 2009; Morin et al., 2007a,b,c; Gebremedhin et al., 2008; Godlewski et al., 2009; Lai et al., 2009; Wang et al., 2011a,b; Enyeart and Enyeart, 2013; Harris et al., 2013; Latorre and Contreras, 2013; Hoshi et al., 2013a,b,c,d; Kacik et al., 2014; Martín et al., 2014; Olszewska et al., 2014; Yan et al., 2014). e, (Chyb et al., 1999; Watanabe et al., 2003; Kahn-Kirby et al., 2004; Hu et al., 2006; Jörs et al., 2006; Oike et al., 2006; Reiter et al., 2006; Andersson et al., 2007; Hartmannsgruber et al., 2007; Matta et al., 2007; Vriens et al., 2007; Rock et al., 2008; Delgado and Bacigalupo, 2009; Shimizu et al., 2009; Parnas et al., 2009a,b; Zhang et al., 2010; Bavencoffe et al., 2011; Motter and Ahern, 2012; Shah et al., 2012; Sukumar et al., 2012; Zheng et al., 2013; Redmond et al., 2014; Ruparel et al., 2015). f, (Fogle et al., 2007; Verkerk et al., 2009). g, (Honen et al., 2003; Woolcott et al., 2006; Muslikhov et al., 2014). h, (Mochizuki-Oda et al., 1993; Asano et al., 1997; Liu et al., 2006; Gutla et al., 2012). i, (Linden and Routtenberg, 1989; Wieland et al., 1992, 1996; Fraser et al., 1993; Charpentier et al., 1995; Kang et al., 1995, 1997; Xiao et al., 1995, 1998, 2000, 2001, 2004, 2005, 2006; Kang and Leaf, 1996; Vreugdenhil et al., 1996; Bendahhou et al., 1997; Fyfe et al., 1997; Macleod et al., 1998; Lee et al., 1999, 2002; Leifert et al., 1999; Ding et al., 2000; Harrell and Stimers, 2002; Leaf et al., 2002; Hong et al., 2004; Jo et al., 2005; Kim et al., 2005; Isbilen et al., 2006; Pignier et al., 2007; Duan et al., 2008; Dujardin et al., 2008; Gu et al., 2009, 2015; Nakajima et al., 2009, 2010; Fang et al., 2011; Guo et al., 2012; Wolkowicz et al., 2014; Safrany-Fark et al., 2015; Wannous et al., 2015). j, (Keyser and Alger, 1990; Finkel et al., 1992; Hallaq et al., 1992; Huang et al., 1992; Shimada and Somlyo, 1992; Damron and Bond, 1993; Dettbarn and Palade, 1993; Pepe et al., 1994; Törnquist et al., 1994; Williams et al., 1994; Roudbaraki et al., 1995; Nagano et al., 1995b; Schmitt and Meves, 1995; van der Zee et al., 1995; Petit-Jacques and Hartzell, 1996; Shimasue et al., 1996; Shuttleworth, 1996; Uehara et al., 1996; Unno et al., 1996; Damron and Summers, 1997; Munaron et al., 1997; Striggow and Ehrlich, 1997; Xiao et al., 1997; Hazama et al., 1998; Chen et al., 1999, 2001; Fang et al., 1999; Liu and Rittenhouse, 2000, 2003; Vellani et al., 2000; Zhang et al., 2000; Barrett et al., 2001; Bringmann et al., 2001; Fiorio Pla and Munaron, 2001; Hirafuji et al., 2001; Krutetskaia et al., 2001; Liu et al., 2001, 2004, 2008, 2015; Luo et al., 2001; Mignen and Shuttleworth, 2001; Ferrier et al., 2002; Soldati et al., 2002; Swan et al., 2003; Yagami et al., 2003; Guermouche et al., 2004; Guibert et al., 2004; Oz et al., 2004; Talavera et al., 2004; Danthi et al., 2005; Erriquez et al., 2005; Rychkov et al., 2005; Yang K. T. et al., 2005; Chemin et al., 2007; Holmes et al., 2007; Liu, 2007; Feng et al., 2008; Rimmerman et al., 2008; Barbara et al., 2009; Heneghan et al., 2009; Mitra-Ganguli et al., 2009; Roberts-Crowley and Rittenhouse, 2009, 2015; Rocha and Bendhack, 2009; DeCostanzo et al., 2010; Cazade et al., 2014; Cui et al., 2014; Thompson et al., 2014). k, (DeCoursey and Cherny, 1993; Kapus et al., 1994; Gordienko et al., 1996; Lowenthal and Levy, 1999; Hourton-Cabassa et al., 2002; Morgan et al., 2002, 2007; Kawanabe and Okamura, 2016)*.

### Historical notes from 1981–1992

In 1981, Takenaka et al. reported that fatty acids with chain lengths exceeding eight carbons, in the concentration range of 0.2–2.2 mM, decreased the voltage-gated Na current in squid giant axons while leaving the delayed-rectifier K current unaffected. *Cis*-2-decenoic acid, which has ten carbons and a double bond between carbon 2 and 3 was the most effective fatty acid in their experiments (Takenaka et al., 1981). In 1987, the same group reported that both saturated and unsaturated medium-chain fatty acids (8–13 carbons) reversibly attenuated voltage-dependent Na currents in squid giant axons by shifting the conductance-vs.-voltage, *G*(*V*), curve in a positive direction along the voltage axis (Takenaka et al., 1987). The effect developed much faster upon intracellular application, suggesting an intracellular site of action. The fatty acid concentration needed for 50% reduction of the peak Na current decreased by a factor of 1/3 for each extra carbon. The presence of a carboxyl or hydroxyl group at the ω end of the fatty acid abolished the effect completely. These findings suggested that a hydrophobic interaction between the fatty acid and Na channel could be an important factor for the effect.

Longer chain fatty acids like palmitic acid (16:0), linoleic acid (18:2), and linolenic acid (18:3) decreased both Na and K currents, but the effects were irreversible, probably because of high concentrations tested would result in micelle formation. Finally, in 1988, by using α-cyclodextrin to dissolve the fatty acids, this group reported that long-chain PUFAs produced effects similar to medium-chain fatty acids (Takenaka et al., 1988). Intracellularly applied AA (20:4) reversibly suppressed the Na current of the squid giant axon with little effect on the K current. 180 μM AA reduced the Na current by 50%, which is a concentration almost ten times lower than required for the medium-chain fatty acid, 2-decenoic acid. Longer PUFAs, Docosatetraenoic (22:4) and DHA (22:6), had effects quantitatively similar to AA. Shorter PUFAs, linoleic acid (18:2) and linolenic acid (18:3), had smaller effects than AA, while the effects of the MUFA oleic acid (18:1) were even smaller, and the SFA stearic acid (18:0) had almost no effect.

In 1989, Bregetovski et al. reported that 2-decenoic acid increased the open probability of K_Ca_ channels up to 10-fold in the membrane of smooth muscle cells from the human aorta (Bregestovski et al., 1989). They suggested that 2-decenoic acid alters the Ca^2+^-binding mechanism of the channel. The same year Linden and Routtenberg reported that low concentrations (1–50 μM) of the MUFA oleic acid (18:1), the PUFAs linoleic acid (18:2), and linolenic acid (18:3), but not the SFA stearic acid (18:0) or the trans-isomer of oleic acid blocked the Na current in N1E-115 neuroblastoma cells (Linden and Routtenberg, 1989); 5 μM oleic acid decreased the peak Na current by 36%. K currents were not affected while both T-type and L-type Ca currents were blocked. This study also excluded the possible explanation that fatty acid effects were produced by increased fluidization of the membrane.

In 1991, Rouzaire-Dubois et al. showed that several MUFAs and PUFAs induced or accelerated inactivation of K_V_ channels via a direct mechanism (not activation of protein kinase C). For instance, 5 μM of oleic acid accelerated the inactivation by a factor of about 10. Among the 18-carbon fatty acids, linoleic acid (18:2) was the most potent inactivator (50-fold acceleration at 5 μM), followed by oleic acid (18:1), linolenic acid (18:3), elaidic acid (18:1, trans), and stearic acid (18:0) which did not affect the inactivation time course at all.

In 1992 several papers on different ion channels established that low μM concentrations of PUFAs affect voltage-gated ion channels, opening as well as closing (Béhé et al., 1992; Finkel et al., 1992; Hallaq et al., 1992; Huang et al., 1992; Kirber et al., 1992; Ling et al., 1992; Shimada and Somlyo, 1992; Wieland et al., 1992).

At about the same time several influential studies were published suggesting that PUFA or PUFA-metabolites had direct effects on other, non-voltage-gated, ion channels (Buttner et al., 1989; Giaume et al., 1989; Kim and Clapham, 1989; Kurachi et al., 1989; Ordway et al., 1989; Anderson and Welsh, 1990; Cantiello et al., 1990; Hwang et al., 1990; Kim and Duff, 1990).

### General effects

Despite the multiple different types of ion channels and PUFAs included in this review, the effects PUFAs have on voltage-gated ion channels are surprisingly general and can be summarized in a few points (Table 1). However, it should be noted that quantitative differences do exist.

**Figure 4:**
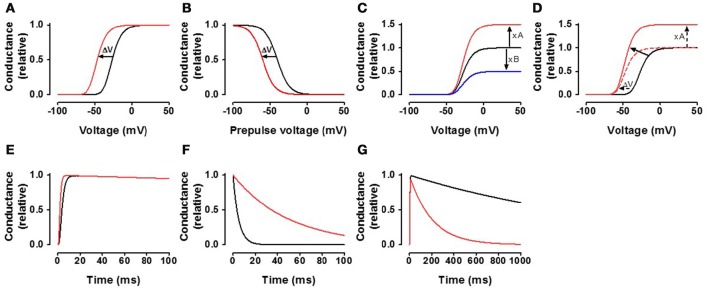
**General effects of the fatty acids on the channels. (A)** The black curve represents a typical control conductance-vs.-voltage curve [*G*(*V*) = 1/(1+exp((*V*-*V*)/s))^*n*^, where *V* is the membrane voltage, *s* = 8 mV, *V* = −40 mV, *n* = 4] for a voltage-gated ion channel. The red curve is the control curve shifted by −20 mV. **(B)** The black curve represents a typical steady-state inactivation curve [*G*(*V*_PP_) = 1/(1+exp((*V*-*V*)/s)), *s* = −8 mV, *V* = −40 mV]. The red curve is the control curve shifted by −20 mV. **(C)** The black curve represents a typical control curve as in **(A)**. The red curve is the control curve increased by a factor 1.5. The blue curve is the control curve decreased by multiplying by 0.5. **(D)** The black curve represents a typical control curve as in **(A)**. The red continuous curve is an example where the curve is both shifted in negative direction along the voltage axis and increased. The amplitude increase can reliably be measured at high voltages where the conductance levels out. The shift can reliably be measured at the foot of the conductance curve (at 10% of the max value of the control curve) without normalization of the curve. The shift of the curve is −20 mV. Measured at the foot, when the maximum conductance is increased by 50%, the shift is over-estimated by 1.7 mV (–21.7 mV instead of –20 mV). **(E)** The black curve represents a typical activation time course (τ = 2 ms, *n* = 4, τ_inact_ = 2 s). The red curve is a two fold increase in opening rate. **(F)** The black curve represents a typical single exponential channel closure (τ = 5 ms). The red curve is 10 times slower. **(G)** The black curve represents a typical channel inactivation (τ = 2 s), while the red inactivates 10 times faster.

- Alteration in voltage dependence of ion channels: A common finding is that PUFAs shift the *G*(*V*) and/or the steady-state inactivation curves in a negative direction along the voltage axis (Figures 4A,B). Such a shift of the *G*(*V*) curve opens the channel, while this shift of the steady-state inactivation curve closes (inactivates) the channel. For Na_V_ and Ca_V_ channels, shifts of the steady-state inactivation curve tend to be larger than shifts of the *G*(*V*) curves. As a consequence, Na_V_ and Ca_V_ channels are generally inhibited by PUFAs. In contrast, K_V_ channels which in many cases are less affected by steady-state inactivation at resting voltage are typically activated by PUFAs.
- Alteration in maximal conductance of ion channels: PUFAs are also able to increase or decrease the conductance at positive voltages (either open probability or the single-channel conductance), where the conductance is not affected as a consequence of the *G*(*V*) shift (Figure 4C). In many cases, there is a combination of effect i and ii (Figure 4D). Despite these combined effects it is relatively easy to distinguish them without curve fitting. Increased conductance can be measured directly at voltages where the conductance has saturated while a *G(V)* shift can be measured at the foot of the curve (e.g. at 10% of maximal conductance in control–the error for the *G*(*V*) curve shown in Figure 4D is only 1.7 mV if the maximal conductance is increased by 50%).
- Alteration in the time course of ion channel kinetics: Consistent with the negative shift of the channel's voltage dependence in negative direction along the voltage axis, the opening kinetics are sometimes faster (Figure 4E) and the closing kinetics slower (Figure 4F) in the presence of PUFAs. There are also multiple reports of a PUFA-induced acceleration of channel inactivation (Figure 4G).

### Specific effects—family by family

Although many of the PUFA effects are general for the voltage-gated ion channels, there are quantitative and qualitative differences. We will therefore briefly describe the specific PUFA effects on different sub-families of voltage-gated ion channels. Table 1 describes the general effects for the specific families and lists the references.

#### K_V_ channels

The largest and most studied family when it comes to PUFA effects on voltage-gated ion channels is the family of voltage-gated K (K_V_) channels. Because of the size and diversity of this family, we will divide this family into three groups of subfamilies. Subfamilies that are not included in our description below have not, to our knowledge, been studied with respect to PUFAs.

K_V_1–4: K_V_ channels within these subfamilies open rapidly and thereby cause fairly fast repolarization of the action potential. Therefore, these channels have special importance for neurons that fire with high frequency. Some of these channels [such as K_V_4 channels which generate transient outward (I_to_) neuronal and cardiac K currents] also inactivate rapidly and are thus sometimes referred to as A-type K_V_ channels. Other members within this subfamily, such as Kv2.1, inactivate slowly generating persistent K currents, in the physiological time frame. Some studies describe PUFA-induced increases in native K currents of unclear molecular identity (e.g., Horimoto et al., 1997; Ferroni et al., 2003; Fioretti et al., 2004), however the most commonly observed PUFA effect on fast native K currents (Lynch and Voss, 1994) and heterologously expressed K_V_1–4 channels is inhibition (by 20–100% at ~10 μM PUFA). This inhibition is commonly associated with an acceleration of the time course of channel inactivation. PUFA effects on channel voltage dependence are less consistent, but the most commonly described are negative voltage shifts of *G*(*V*) and/or steady-state inactivation curves. The overall effect is typically a reduced current, but a few exceptions describe PUFA-induced activation of K_V_1–4 channels (Zhao et al., 2007; Börjesson et al., 2008, 2010; Zhang M. et al., 2008; Börjesson and Elinder, 2011).

K_V_7: K_V_ channels within this subfamily open slowly and are referred to as slow delayed rectifiers. K_V_7 channels underlie the neuronal M current, which contributes to the negative resting membrane potential in neurons, and the cardiac I_Ks_ current, which contributes to the repolarization in cardiomyocytes. PUFAs are reported to activate both natively and heterologously expressed K_V_7 channels. PUFA-induced increases of K_V_7 current amplitudes are associated with a small negative shift in the *G*(*V*) curve (roughly −5 to −10 mV by 10 μM PUFA). There are, however, some inconsistencies concerning the role of the auxiliary subunit KCNE1 during PUFA exposure. The cardiac I_Ks_ channel is a complex between K_V_7.1 and KCNE1. Doolan et al. find that PUFA effects on the I_Ks_ channel require the presence of KCNE1 (Doolan et al., 2002). In contrast, we describe that KCNE1 causes reduced PUFA sensitivity of the I_Ks_ channel compared to K_V_7.1 alone (Liin et al., 2015). Moreover, Moreno *et al*. show that PUFA effects on the I_Ks_ channel vary over time (Moreno et al., 2015).

K_V_10–12: These subfamilies contain the K_V_10.1 channel (= EAG1) and the K_V_11.1 channel (= hERG or ERG1). K_V_11.1 forms the major portion of the rapid delayed rectifier current (I_Kr)_, which is critical in correctly timing the repolarization of cardiac action potentials. Mutations in K_V_11.1 and compounds targeting I_Kr_ channels can cause long QT syndrome and subsequent lethal ventricular fibrillation. Most PUFA studies on this group have been performed on the K_V_11.1 channel, with a single study performed on K_V_10.1. The effects in this small group are mixed. Both current reductions and current increases have been reported. The *G*(*V*) curve is negatively shifted in most studies. This shift is rather large for K_V_10.1, around −30 mV at 10 μM for all PUFAs studied (Gavrilova-Ruch et al., 2007). Several studies also suggest that PUFAs speed up closure (inactivation) of these channels.

#### K_Ca_ channels

The family of Ca-activated K channels contains three types of channels: Big, intermediate, and small conductance channels. Only the K_Ca_1.1 (BK) family is clearly voltage dependent as it is opened by alterations in membrane voltage *in addition to* increases in the intracellular Ca^2+^ concentration. Almost all studies of PUFA effects on K_Ca_ channels have been performed on K_Ca_1.1 channel. This channel is essential for the regulation of smooth muscle tone and neuronal excitability. PUFAs, even at submicromolar concentrations, increase the maximum conductance *and* shift the *G*(*V*) curve in negative direction along the voltage axis. In addition, the K_Ca_1.1 channel is quite sensitive to PUFA metabolites (Meves, 2008). Recent studies have mapped the binding site for PUFAs to a region near the intracellular gate (Hoshi et al., 2013d; Tian et al., 2016).

#### TRP channels

The transient receptor potential (TRP) channels form a large family, consisting of 28 channels divided in six subfamilies. TRP channels are for example involved in mediating the sensations of cold, heat, and pain. These channels are fairly non-selective and therefore conduct several types of cations (e.g., Na^+^, Ca^2+^). TRP channels are generally described as being activated by PUFAs. However, many of these studies measured TRP channel activity indirectly using fluorescence-based calcium imaging, which provides limited information about TRP channel voltage dependence and the time course of TRP currents. In studies that include electrophysiological recordings (primarily from TRPVs, TRPCs, TRPAs, and *drosophila* TRPs), the amplitude of TRP currents are found to increase many-fold following application of >10 μM PUFA. Moreover, Shimizu et al. describe a PUFA-induced negative shift in the *G*(*V*) curve of TRPP3 channels (Shimizu et al., 2009). However, TRPM channels are an exception among TRP channels, as they are almost completely inhibited by PUFAs (Andersson et al., 2007; Parnas et al., 2009b; Bavencoffe et al., 2011).

#### Na_V_ channels

The family of voltage-gated Na channels contains the first ion channel to be discovered and explored electrophysiologically (Hodgkin and Huxley, 1952a,b), and later, cloned and sequenced (Noda et al., 1984). Na_V_ channels generate action potentials in neurons, the heart, and other muscles. Thus, they are important targets for the regulation of excitability. With few exceptions, PUFAs reduce Na_V_ currents. However, PUFAs also shift the *G*(*V*) and steady-state inactivation curves of most Na_V_ channels in a negative direction along the voltage axis. In general, the steady-state inactivation curve is shifted more than the *G*(*V*) curve. These shifts have conflicting results; the *G(V)*-curve shift opens channels and thereby increase excitability, while the steady-state inactivation curve shift inactivates/closes channels and thereby decrease excitability. Altogether, these mixed effects result in reduced excitability.

#### Ca_V_ channels

Voltage-gated Ca channels have two critical functions: Generating (or boosting) action potentials, and conducting extracellular Ca^2+^ ions into the cell where they can act as a second messenger. PUFA effects on Ca_V_ channels have been studied rather extensively. The effects are very similar to the effects on Na_V_ channels, that is, the maximal conductance is decreased, and *G*(*V*) and steady-state inactivation curves are shifted in a negative direction along the voltage axis, with the steady-state inactivation shift being larger than the *G*(*V*) shift. In addition, the inactivation time course is in some cases accelerated. Altogether, these mixed effects result in reduced excitability.

#### H_V_ channels

The proton channel, which was cloned only 10 years ago (Ramsey et al., 2006; Sasaki et al., 2006), deviates from all other ion channels in lacking the conventional ion-conducting pore domain. However, the voltage sensing mechanism is similar to the other voltage-gated ion channels; the difference is that two VSDs act together as a dimer (Koch et al., 2008; Lee et al., 2008). The effects of PUFAs on the H_V_ channels are reminiscent of the effects on the other channels, suggesting that at least some of the effects are conferred by the VSD. PUFAs increase the maximal current of H_V_ channels–for most other channels the maximal current is decreased. The shift of the *G*(*V*) is in the negative direction along the voltage axis, but the size is smaller than for most other channels. One surprising finding is that the PUFA carboxyl charge is not important for this effect (Kawanabe and Okamura, 2016).

#### Other voltage-gated ion channels

Several other ion channels belonging to the superfamily of voltage-gated ion channels have been explored with respect to PUFA effects, but many of them are difficult to study in biophysical detail. For several of the families only few studies have been performed, often with mixed data, making it difficult to draw general conclusions. These families are briefly mentioned here and the references are found in Table 1. The family of cyclic-nucleotide gated (CNG) ion channels contains two types of channels–hyperpolarization-activated cyclic nucleotide gated (HCN) channels, which are highly voltage dependent (even though the polarity is opposite to most other ion channels), and the non-voltage dependent CNG channels. HCN channel have an important role as pacemaker channels in the sino-atrial node of the heart. AA has been found to directly facilitate HCN channel opening, and rats fed a diet enriched with fish oil show reduced pacemaker currents and consequently reduced heart rate (see Table 1). The ryanodine receptor (RyR) family is an intracellular cation channel critical for the regulation of intracellular levels of Ca^2+^. PUFAs have been reported both to increase and decrease the RyR current. CatSper channels and TCP channels are molecularly related. CatSper channels are found in the plasma membrane of sperm while TCP channels are found in intracellular endolysosomes. Here the effects of PUFAs are also mixed.

## Sites and mechanisms of actions of PUFA

There are some general properties of fatty acids that are often described as being required to induce the PUFA effects described above (e.g., Xiao et al., 1997, 1998; Danthi et al., 2003, 2005; Börjesson et al., 2008; Liin et al., 2015):

- At least two double bonds in the acyl tail are required. Therefore, PUFAs induce these effects while SFAs and MUFAs generally do not. However, there is usually no clear difference between n-3 and n-6 PUFAs. Also, there is no large or systematic difference between PUFAs with respect to chain lengths from 16 to 24 carbons.
- *Cis*-geometry of the double bonds in the acyl tail is required. *Trans*-geometry renders the PUFAs ineffective.
- The negative charge of the carboxyl group is required. Uncharged methyl esters of PUFAs generally lack effects.

In addition, PUFAs need to remain in their intact form. Experiments conducted with non-metabolizable PUFA analogs (such as ETYA) and cyclooxygenase inhibitors (that prevent PUFA metabolism) show that the PUFAs themselves, and not their metabolites, induce these general effects. Some exceptions, however, have been reported (Twitchell et al., 1997; Lee et al., 2002; Judé et al., 2003).

Despite the large number of studies published (Table 1), only a few PUFA sites of action have been described and little has been described concerning the mechanism by which PUFAs interact with voltage-gated ion channels.

The first major question is whether the reported effects of PUFAs on the voltage-gated ions channels are direct channel effects or if they are mediated via non-specific membrane effects. In general, the concentrations needed for the PUFA effects are relatively low (1–10 μM), ruling out unspecific membrane fluidizing effects (Pound et al., 2001). Moreover, there is no correlation between a PUFA's propensity to fluidize the membrane and their effects on voltage-gated ion channels (Villarroel and Schwarz, 1996). Alterations of the lipid membrane by soaking out cholesterol affect ion channel function but do not affect acute PUFA effects (Moreno et al., 2015). Further, the onset and washout of the effect on K_V_ channels is very rapid (2–3 s), suggesting a direct channel effect (Poling et al., 1996). An early suggestion that PUFAs may bind directly to voltage-gated ion channels came from experiments on Na_V_ channels in which the PUFA eicosapentaenoic acid (EPA) inhibited the binding of a radio-labeled toxin to cardiac Na_V_ channels (Kang et al., 1995; Kang and Leaf, 1996). Further evidence that PUFAs have direct ion channel effects is provided by the demonstration that single point mutations in various voltage-gated ion channels also affects the ability of PUFAs to modulate those channels (e.g., Xiao et al., 2001; Börjesson and Elinder, 2011; Ottosson et al., 2014; Liin et al., 2015).

Secondly, we may ask on which side of the membrane the PUFAs act. Whereas, most studies have used extracellular application of PUFAs, one study made a direct comparison of PUFA-induced effects upon PUFA application from either side of the membrane. They found no difference in PUFA effects on K_V_ channels based on the side of application (Oliver et al., 2004). In contrast, some studies have demonstrated ion channel modulation when PUFAs are applied extracellularly but fail to observe modulation when PUFAs are added intracellularly (Honoré et al., 1994; Poling et al., 1995, 1996; Garratt et al., 1996; Kehl, 2001; McKay and Jennings, 2001; Guizy et al., 2008). Yet other studies primarily observe effects when the PUFAs are applied to the intracellular side (Boland et al., 2009; Decher et al., 2010). These differences in the side of action may be explained by differences in the predominant PUFA sites of action in different types of ion channels.

### Five sites of action

**Figure 5:**
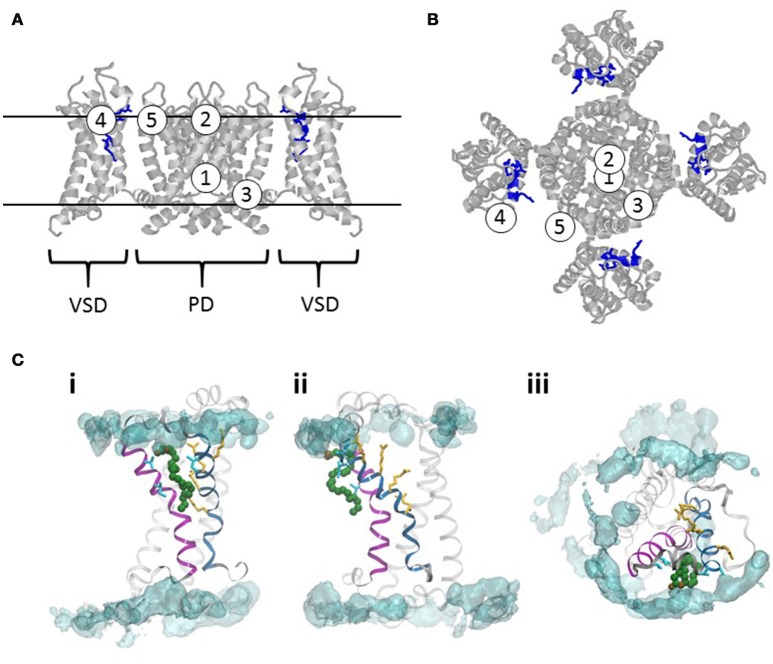
**Sites of actions of PUFAs on voltage-gated ion channels. (A)** A homology model of the Shaker K_V_ channel based on the structure of the K_V_2.1/1.2 chimera (Long et al., 2007; Henrion et al., 2012). Side view. VSD denote one voltage-sensor domain. PD denotes the pore domain. For clarity, the VSD in the front and the back are removed. The long loop between S3 and S4 are removed (residues 337–353). The two continuous lines delineate the approximate outer and inner borders of the lipid bilayer. The Figures 1–5 denote five proposed sites of actions of PUFA. **(B)** Top view of the channel in **(A)**. **(C)** Interaction site for a DHA molecule with the VSD of the Shaker K_V_ channel. The helix in magenta is S3 and the helix in blue is S4. The four yellow amino acid residues are the four gating charges [R362 (in the top), R365, R368, and R371]. The four residues in cyan (two in S3, residues 325 and 329; two in S4, 359 and 360) are the residues identified to be close to the PUFA binding site (Börjesson and Elinder, 2011). A typical binding pose for a DHA molecule in green is from Yazdi et al. (2016). The POPC lipid bilayer is represented by a cyan iso-density surface corresponding to the positions of lipid nitrogens in the simulation at 5% occupancy. The left and middle panels are the VSD viewed along the membrane from two different angles. The right panel is the VSD viewed from the extracellular side.

From our analysis of PUFA publications in the field we have identified five sites of actions (Figures 5A,B). The first two sites are located in the ion-conducting pore, one at the intracellular entrance (PUFA site 1), and the other at the extracellular entrance (PUFA site 2). The third is located at the VSD-to-pore domain linker close to the intracellular gate (PUFA site 3). The last two are located at the interface between the extracellular part of the ion channel and the outer leaflet of the lipid bilayer from which PUFAs electrostatically interact with the VSD (PUFA site 4) or the pore domain (PUFA site 5).

#### PUFA site 1–the intracellular cavity

Several studies have identified the intracellular part of the pore lining S6, with residues facing the intracellular cavity, as critical for the PUFA effects. A common mechanism is an open-channel block causing a time dependent current reduction–an inactivation.

A single point mutation of domain I of the cardiac Na_V_1.5 channel (N406K) clearly reduces the inhibitory effect of DHA (Xiao et al., 2001). The negative shift of the steady-state inactivation curve is also attenuated. The identified amino-acid residue is located in the middle of S6, facing the intracellular cavity, in a similar position where local anesthetics bind to domain IV of a rat brain Na_V_ channel (Ragsdale et al., 1994). However, the molecular detail why the steady-state inactivation curve is shifted by DHA has not been described.

In K_V_1.1 channels, DHA and AA, but also the uncharged anandamide induces inactivation by interacting with hydrophobic residues lining the inner cavity of the pore (Decher et al., 2010). The inactivation was suggested to be caused by open-channel block by PUFA binding to the cavity of the channel. K_V_1.5 has been proposed to be inactivated via a similar mechanism. Point mutations combined with computer docking support PUFA binding in the cavity (Bai et al., 2015).

In the Ca-activated K_Ca_3.1 (= SK4 or I_K1_) channel, which is not voltage sensitive despite having VSDs, AA inhibits the current. This inhibition is completely prevented by the T250S mutation at the inner end of the pore loop, together with the V275A mutation in the middle of S6, close to residue 250 (Hamilton et al., 2003). Furthermore, introducing the threonine and the valine in the equivalent positions of the AA-insensitive K_Ca_2.2 (= SK2) channel makes this channel sensitive to AA. Thus, AA interacts with the pore-lining amino acids of K_Ca_3.1 to inhibit the channel.

Thus, several studies on different ion channels have identified the middle of S6, in the cavity, as a major determinant for PUFA interactions.

Another type of channel-inactivating pore-interacting mechanism has been described for AA on K_V_3.1 (Oliver et al., 2004). AA is equally effective from either side of the membrane. AA-induced inactivation was not affected by the presence of TEA at the extracellular or intracellular side of the channel protein. These results rule out open-channel block as the mechanism underlying AA-induced inactivation, but suggest a lipid-induced closure of the “pore gate”.

#### PUFA site 2–the extracellular entrance of the ion conducting pore

K_V_1.1 (Garratt et al., 1996), K_V_1.2 (Garratt et al., 1996; Poling et al., 1996), K_V_1.5 (Honoré et al., 1994; Bai et al., 2015), and K_V_3.1a (Poling et al., 1996) are inactivated by PUFAs via a proposed open-channel block where the pore is accessed from the extracellular side. Point mutations combined with computer-guided docking support a PUFA binding site at the extracellular entrance of the pore (Bai et al., 2015).

#### PUFA site 3–the intracellular gate (lower end of S6 and S4—S5 linker)

Some studies have identified a PUFA site at the inner end of S6 or in the S4–S5 linker, which are close to each other and form the intracellular gate of the channel (Long et al., 2005). In the absence of detailed data we have brought them together to a single site. The difference from PUFA site 1 and 2 is that this site is outside the central axis of the channel and that this site thus can host PUFA molecules to open the channel by bending the gate open.

The Ca^2+^-activated K_Ca_1.1 (= BK) channel is, in contrast to the Na_V_1.5 and the K_Ca_3.1 channels described above, opened by several PUFAs such as DHA, AA and α-linolenic acid. Hoshi and collaborators have identified Y318 near the cytoplasmic end of S6 in the K_Ca_1.1 channel as a critical determinant of the stimulatory action of DHA (Hoshi et al., 2013d; Tian et al., 2016). The Y318S mutation greatly diminishes the channel's response to DHA, but not to AA or α-linolenic acid.

K_V_4.2 inactivates very quickly upon application of AA, while the inactivation of the Shaker K_V_ channel is fairly unaffected. Transplanting the Shaker S4–S5 linker to K_V_4.2 attenuates the effect of AA on the K_V_4.2 channel, and conversely, transplanting the K_V_4.2 S4–S5 linker to the Shaker K_V_ channel makes the Shaker K_V_ channel more sensitive to AA (Villarroel and Schwarz, 1996). Molecular docking approaches using a K_V_4.2 homology model predicted a membrane-embedded binding pocket for AA comprised of the S4–S5 linker on one subunit and several hydrophobic residues within S3, S5, and S6 from an adjacent subunit (Heler et al., 2013). The pocket is conserved among K_V_4 channels.

#### Pufa site 4–lipoelectric effects on S4 charges of the voltage-sensor domain

It is well-known that the lipid environment is important for the function of voltage-gated ion channels. Crystal structures show that phospholipids are making close and specific contacts with the channel (Long et al., 2007). Molecular dynamics simulations suggest that the negatively charged phosphate group of phospholipids make electrostatic interactions with the positive charges of the voltage sensor (Freites et al., 2005; Sansom et al., 2005). Experiments altering the charge of the phospholipids show that the charge of the phospholipids is necessary for proper function of voltage-gated ion channels (Schmidt et al., 2006). Free PUFA molecules can also affect ion-channel gating. PUFA molecules in the extracellular solution can quickly incorporate in the extracellular leaflet of the phospholipid bilayer; the hydrophobic tail is tucked into the hydrophobic part of the bilayer and the carboxyl group is facing the extracellular water (Feller et al., 2002; Yazdi et al., 2016). The PUFA molecules are most likely everywhere in the lipid bilayer but they could potentially be clustered around ion channels (Yazdi et al., 2016).

In studies of the Shaker K_V_ channel and several K_V_7 channels we have identified a site between the extracellular leaflet of the lipid bilayer and S4 of the VSD. Mutational analysis and molecular dynamics simulations have suggested that the PUFA molecules interact between the transmembrane segments S3 and S4 and the lipid bilayer (Figure 5C) (Börjesson and Elinder, 2011; Yazdi et al., 2016). The electric charge of free PUFA molecules in the lipid bilayer affects the gating machinery of the VSD (Börjesson et al., 2008, 2010; Börjesson and Elinder, 2011; Ottosson et al., 2014; Liin et al., 2015, 2016b; Yazdi et al., 2016). Because lipophilicity and electrostatic forces are central in this model, we have called this the *lipoelectric* mechanism.

#### Pufa site 5–lipoelectric effects on the pore domain

PUFAs modulate the K_V_1.4 channel inactivation. It has been suggested that the PUFA molecule partition in the membrane as has been suggested for PUFA site 4. The difference is that the negatively charged PUFA molecule line up outside the pore domain and from this position the acidic head group of the PUFAs raises the pK_a_ of H508 in the pore domain. This raised pK_a_ of the histidine reduces the K^+^ occupancy of the selectivity filter, stabilizing the C-type inactivated state (Farag et al., 2016).

### Helical screw and a mechanism by which PUFAs can open an ion channel

Of all five sites described above, the mechanism by which PUFAs affect K_V_ channels via PUFA site 4 has been studied in most detail. In the remaining part of this section we will focus on this PUFA mechanism. The mechanism by which voltage-gated ion channels sense membrane voltage is central for this effect (reviewed for instance in Armstrong, 1981; Keynes and Elinder, 1999; Bezanilla, 2000; Swartz, 2004; Börjesson and Elinder, 2008). Therefore, we will here, in brief, describe the mechanism for voltage sensing.

**Figure 6:**
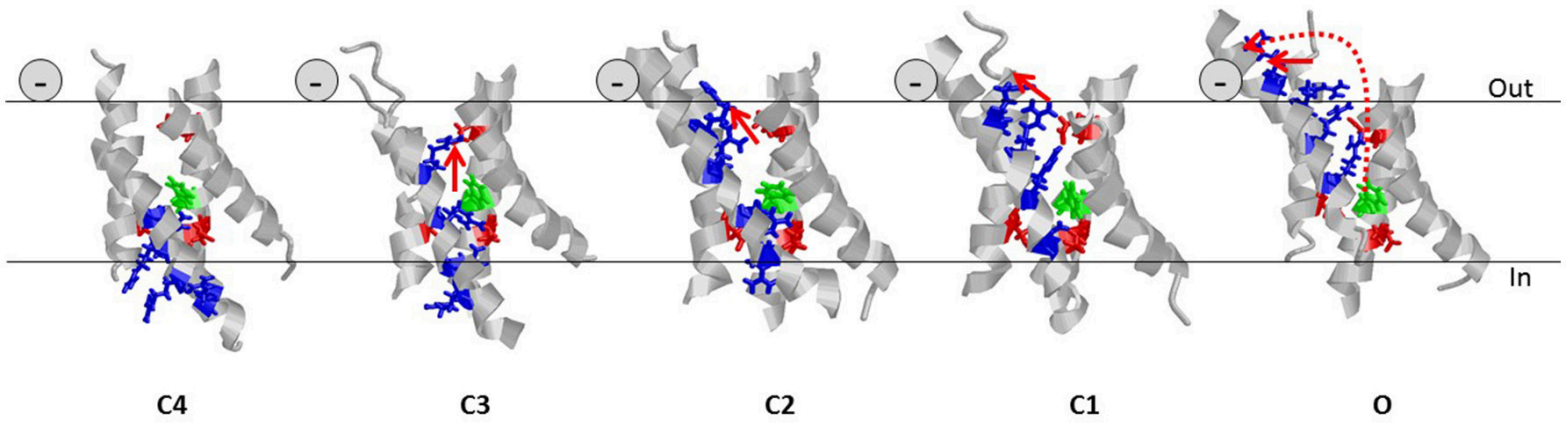
**Helical screw and the lipoelectric effect**. Five states of the VSD of the Shaker K_V_ channel are shown (Henrion et al., 2012). For clarity, only the transmembrane segments (224–246, 278–300, 311–332, 354–377) are shown and the intra- and extracellular loops are removed. Gating charges (residues R362, R365, R368, and R371) of S4 are shown as blue sticks. Negative counter charges (E283, E293, and D316) are shown as red sticks. The hydrophobic barrier in S2 is shown in green (F290). The continuous red arrows indicated the movement of the charge of R362 in each step. The dotted red arrow in state O denotes the complete movement of R362, from state C4 to state O. The negative sign denotes the position of the carboxyl group of the PUFA molecule.

The four VSDs connected to a central ion-conducting pore domain make, in most cases, the channel voltage sensitive. Each VSD has four transmembrane segments labeled S1 to S4. The fourth transmembrane segment, S4, has several positively charged amino-acid residues (blue sticks in Figure 6) interspaced by two hydrophobic residues. The transmembrane segments S1 to S3 host negative counter charges (red sticks in Figure 6) that neutralize the positive S4 charges in the transmembrane section of the VSD. The positive charges of S4 can change partners and thereby slide along the rest of the VSD (from the deepest state C4 to the open state O in Figure 6). At negative membrane voltages, S4 is close to the intracellular side (the down state) and at positive membrane voltages S4 is close to the extracellular side (the up state) of the membrane. At resting states C4 and C3 most S4 charges are below the hydrophobic barrier (Tao et al., 2010) (the green phenylalanine in Figure 6). Upon activation three to four charges of each S4 move across the barrier, in three to four discrete steps. The total movement is around 13 Å, even though distances from 7 to 15 Å have been reported (e.g., Ruta et al., 2005; Campos et al., 2007; Delemotte et al., 2011; Henrion et al., 2012).

S4 not only slides along S1–S3 during activation but also rotates around its longitudinal axis because the positive charges are spiraling around S4 (Figure 6). This means that the top positive charge in S4 (R1) moves in a spiral from the center of the channel to the extracellular surface and then along the surface (arrows in Figure 6). Thus, fixed negative charges at or close to the extracellular surface of the channel can electrostatically “pull” S4 to open the channel, while fixed positive charges could do the opposite. For instance, charged residues in the extracellular linkers connecting the transmembrane segments of a voltage-gated ion channel can control the voltage dependence of the channel (Elinder et al., 2016).

Our data are consistent with one (or several) PUFA molecules interacting with the VSD close to a cleft between the extracellular ends of S3 and S4 (Börjesson and Elinder, 2011). Experimental data from the Shaker K_V_ channel suggests that it is mainly the C1 → O transition that is affected by the PUFA molecules and that the top charge of S4, which moves horizontally along the lipid bilayer during this last step, is the most important charge for the effect.

### Data supporting the lipoelectric model

**Figure 7:**
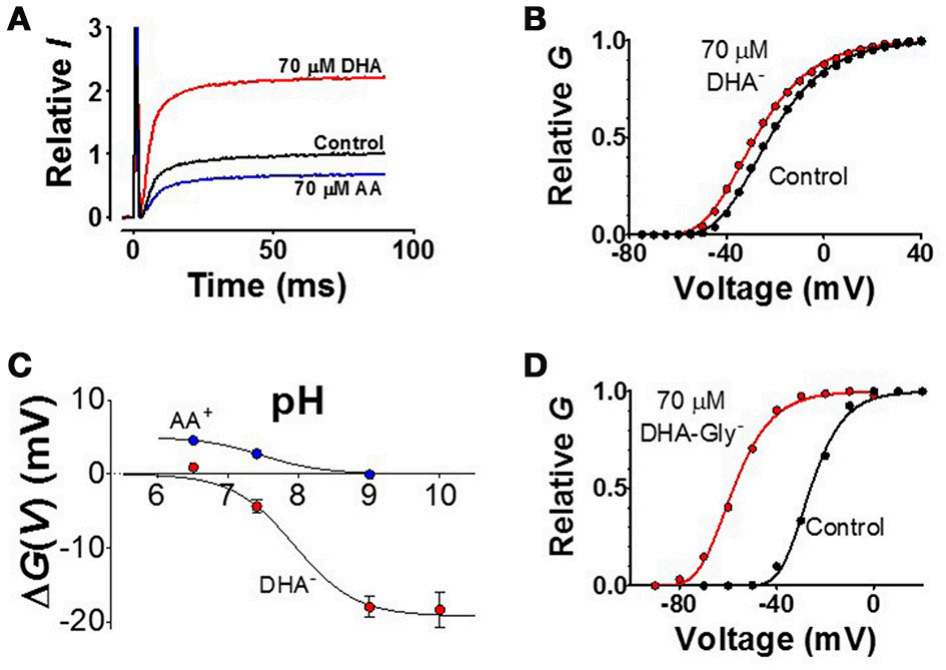
**Examples of experimental data. (A)** K current though Shaker K_V_ channels at a voltage step to V = −40 from a holding voltage of −80 mV. The negatively charged DHA increases the current (red curve), while the positively charged arachidonoyl amine (AA^+^) decreases the current (Börjesson et al., 2010). **(B)** The normalized conductance-vs.- voltage curve for control and 70 μM DHA (Börjesson et al., 2010). **(C)** pH dependent shift of the *G*(*V*) curves (Börjesson et al., 2008, 2010). **(D)** Decreasing the pK_a_ value of the DHA molecule, and thereby charging the molecule, by adding a glycine motif increases the shift (Liin et al., 2015) (data on the K_V_7.1 channel).

**Figure 8:**
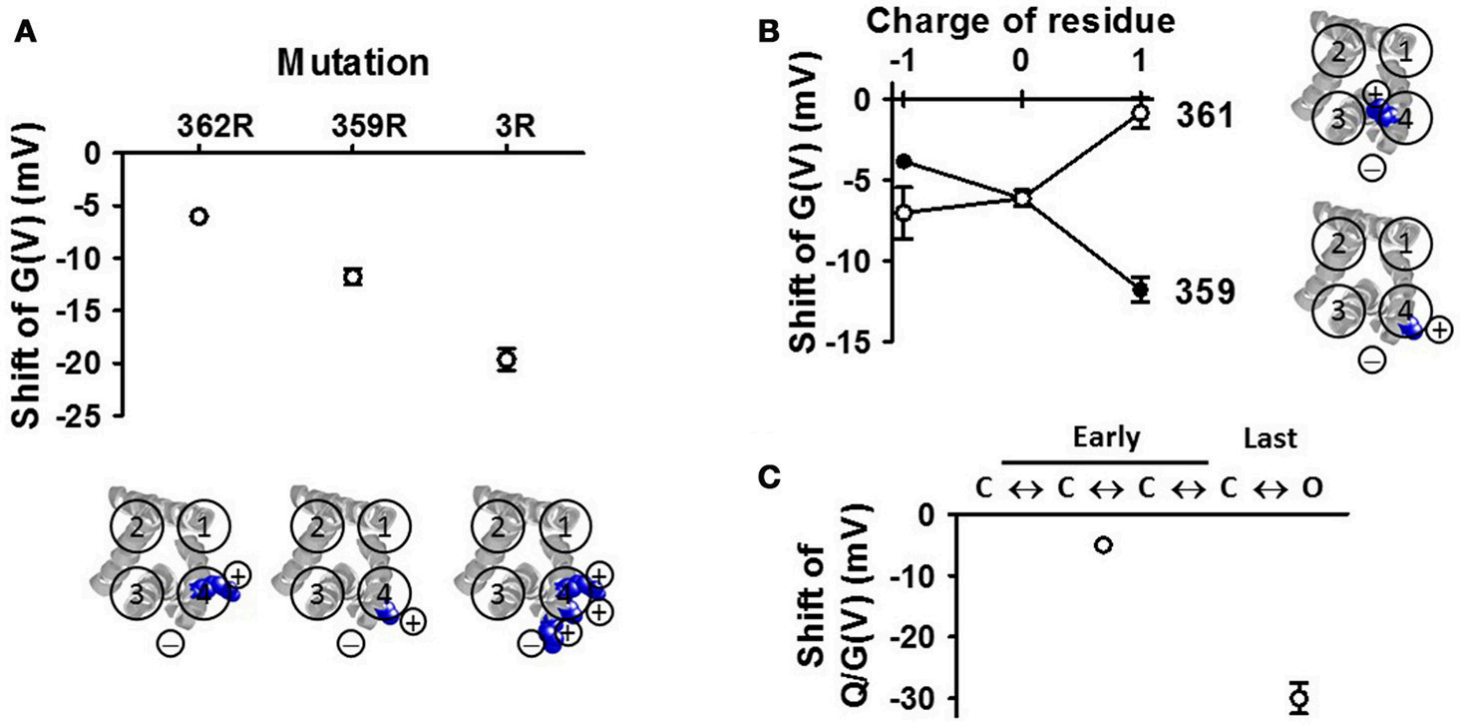
**VSD mutations affect the DHA effect on the Shaker K_V_ channel (A)** The number and positions of the arginines in the top of S4 affects the DHA-induced *G*(*V*) shift (Ottosson et al., 2014). The 3R channel contains the positive charges R356, R359, and R362. Only the transmembrane parts are shown (residues 224–246, 278–300, 311–332, 354–377) **(B)** Altering the valence or altering the side of S4 for the charge alters the effect of DHA (Ottosson et al., 2014). **(C)** Effects on the early and late transitions are measured on the conducting and non-conducting ILT-mutant (Börjesson and Elinder, 2011).

Here we list experimental support for the proposed lipoelectric model. Most of the experiments have been performed on the Shaker K_V_ channel. Some experiments have also been performed on K_V_7.1 and K_V_7.2/3 channels:

1. The sign and size of the PUFA charge is critical for the effect. (i) A PUFA molecule, expected to be at least partially negatively charged at neutral pH, increases the current (Figure 7A, red curve) by shifting the *G*(*V*) curve in negative direction along the voltage axis (Figure 7B, red curve), as expected from an electrostatic mechanism. (ii) If the PUFA molecule is not permanently charged at neutral pH, alterations in pH are expected to affect the PUFA effect. In fact, pH has a pronounced effect on the *G*(*V*) shift for PUFAs (Figure 7C, red symbols). At pH 6.5 there is no shift as if the PUFA molecule is uncharged. At pH 9 or 10 the shift is saturated as if the PUFA molecule is fully negatively charged. The midpoint value of the curve is at pH 7.9 for the Shaker K channel. This surprisingly high value compared to the predicted pK_a_ value in solution of pH 4.9 suggests that the local pH at the surface is radically different from the bulk solution. Similar effects have been described for K_V_7.1 (pK_a_ = 7.7) and K_V_7.2/3 (pK_a_ = 7.5). Alteration of the charge of amino acids close to the binding site can alter the apparent pK_a_ value of PUFAs (Börjesson and Elinder, 2011). Interestingly the auxiliary subunit KCNE1 alters the pK_a_ value of K_V_7.1 to pK_a_ = 8.6 to render the channel essentially insensitive to PUFA at neutral pH (Liin et al., 2015). (iii) If the charge is essential, an uncharged molecule should not shift the *G*(*V*) and a permanently charged should shift the *G*(*V*) as much the PUFA molecule at high pH. In fact, uncharged methyl esters of the PUFAs do not shift the *G*(*V*) despite competing with PUFAs for the same site (Liin et al., 2015). Designed PUFAs with a shifted pK_a_ value, for instance docosahexaenoyl glycine (DHA-Gly), shifts the *G*(*V*) much more than a PUFA molecule at neutral pH (Figure 7D) (Liin et al., 2015, 2016b). Most importantly, a positively charged “PUFA” should shift the *G*(*V*) in positive direction along the voltage axis and reduce the current. This is in fact the case (Figures 7A,C blue trace and symbols) (Börjesson et al., 2010; Liin et al., 2015). Also these positively charged PUFA analogs show pH dependence, but now the effect is in opposite direction (Figure 7C).
2. The positions and valence of the charges on S4 are critical. To investigate the PUFA interaction with S4, in closer detail, we decorated the extracellular end of S4 in the Shaker K_V_ channel with positively charged residues in different positions (Ottosson et al., 2014). The major findings were the following: (i) moving the top charge R1 in S4 from position 362 to 359 (by constructing the A359R/R362Q mutant) increased the effect by DHA by a factor of about two (Figure 8A). (ii) Adding more arginines than just one sometimes increased the effect; adding two extra charges (356R and 359R) to the existing top charge of S4 (R362) increased the PUFA-induced *G*(*V*) shift by a factor of three (Figure 8A). Because there are three (positively charged) arginines in the sequence 356–362 in this construct, we have called this the 3R channel. (iii) A positively charged residue on the opposite side to R359 (the most influential charge) of the α-helical S4 (i.e., R361) abolished the PUFA-induced *G*(*V*) shift (Figure 8B), supporting the idea that S4 rotates and that R1 is moved along the bilayer surface (at least in its last step). (iv) Negatively charged residues introduced at these specific positions in S4 had opposite effects to positive charges supporting electrostatic effects.
3. PUFA mainly act on the final channel-opening step. A voltage-gated ion channel undergoes several voltage dependent transitions between closed (C) states before it enters into the open (O) state (Figure 8C). PUFAs act on the voltage-sensor transitions and can theoretically act on any of the transitions. It is possible to differentiate effects on the early voltage-dependent transitions, before the channel reaches the open state, and the final voltage-dependent transition, which open the channel, in the Shaker K_V_ channel by introducing a set of mutations in S4 (the ILT mutation) (Smith-Maxwell et al., 1998). We found that DHA only has a minor effect on the early transitions, and that almost all effects of DHA are on the last step (Börjesson and Elinder, 2011). This means that the critical, PUFA-sensitive step, is when R1 moves from a position close to the pore domain to a position close to the lipid bilayer (C1 to O in Figure 6). In K_V_7.1 channels, both early S4 movements and S4 movements associated with channel opening are affected by PUFA (Liin et al., 2016b). However, the relative PUFA effect on these different gating transitions in the K_V_7.1 channel remains to be quantified.

### Other properties important for the effect of PUFAs

The lipolelectric mechanism described above clearly explains why the charge of the PUFA molecule plays such an important role. However, we have less information about why multiple double bonds in *cis* geometry in the tail are required, and we have no information about the length of the tail. Double bonds restrict the conformational freedom in C = C bonds of the fatty acid but double bonds in *cis* geometry (Figure 2), causes the chain to bend and explore conformations not found for saturated fatty acids–the more *cis* double bonds the more curved the molecule is. The curvedness goes from a kink for one double bond to hairpin shapes for five or six double bonds. DHA, a PUFA with a 22-carbon chain and six *cis* double bonds, undergoes fast conformational changes and has a highly flexible structure (Eldho et al., 2003). In contrast, double bonds in *trans* geometry (Figure 2) do not cause the chain to bend much, having a shape similar to straight saturated fatty acids. Thus, it is not surprising that saturated fatty acids and *trans* PUFAs lack effects on ion channels if a curved shape is required.

The same pattern for the effective molecules is not restricted to voltage-gated ion channels but also to fatty-acid activation of K_2P_ channels where fatty acid-induced stimulation requires at least one C = C bond and the anionic (COO-) form of the fatty acid (Lotshaw, 2007). Unesterified DHA molecules are predicted to infiltrate certain spaces between the transmembrane helices of rhodopsin (Grossfield et al., 2006). The dynamic changes in the protein during gating would thus be influenced by the packing of DHA within these spaces, which could explain how DHA facilitates conformational changes in rhodopsin upon activation (Feller and Gawrisch, 2005). This flexibility can explain the promiscuity of the PUFAs, why they act on so many channels and sites.

## Physiological and therapeutic concentrations

The concentration of unesterified PUFAs available to affect voltage-gated ion channels in different tissues is largely unknown. It is therefore difficult to assess the physiological relevance of the PUFA effects described in this paper. A concentration range of 1–30 μM of PUFA is often effective for experimental modulation of voltage-gated ion channels. The concentration of unesterified PUFA in plasma has been reported to be roughly 10–50 μM (Burtis and Ashwood, 1998; De Caterina et al., 2000; Fraser et al., 2003; Siddiqui et al., 2008). This plasma concentration of PUFA can dramatically increase to 130–400 μM during consumption of certain diets (Kuriki et al., 2002; Fraser et al., 2003; Siddiqui et al., 2008). Moreover, the local PUFA concentration in specific tissues may be increased during pathological conditions such as ischemia and epileptic seizures (Hochachka, 1986; Siesjö et al., 1989). Based on these reported PUFA concentrations it seems plausible that most voltage-gated ion channels that are PUFA sensitive would experience some degree of PUFA modulation under physiological as well as pathological conditions. The outcome of different synergistic and opposing PUFA modulations in terms of, for instance, neuronal and cardiac excitability is hard to predict and would depend on the PUFA sensitivity and relative importance of each type of ion channel.

## Concluding remarks

In the present review, we have suggested five different PUFA-binding sites. Two of the sites (PUFA site 1 and PUFA site 2) are located in the ion conducting pore and binding to these sites reduce the current. Two of the sites (PUFA site 3 and PUFA site 4) can either increase or decrease the open probability of the channel by either affecting the gate (PUFA site 3) or the voltage sensor (PUFA site 4). Finally, one site in the periphery of the pore domain (PUFA site 5) can regulate slow inactivation by acting on distance. We suggest that all five sites can exist in a single ion channel and the overall effect is determined by the relative contributions of the five sites.

## Author contributions

FE and SL designed the study, analyzed data, and wrote the manuscript.

### Conflict of interest statement

The authors declare that the research was conducted in the absence of any commercial or financial relationships that could be construed as a potential conflict of interest.
